# Commentary: The Influence of Proton Pump Inhibitors on the Fecal Microbiome of Infants with Gastroesophageal Reflux-A Prospective Longitudinal Interventional Study

**DOI:** 10.3389/fcimb.2018.00430

**Published:** 2018-12-14

**Authors:** Kelsea M. Drall, Hein M. Tun, Anita L. Kozyrskyj

**Affiliations:** ^1^Department of Pediatrics, University of Alberta, Edmonton, AB, Canada; ^2^School of Public Health, University of Alberta, Edmonton, AB, Canada; ^3^Department of Obstetrics and Gynecology, University of Alberta, Edmonton, AB, Canada

**Keywords:** proton pump inhibitors, infant gut microbiota, GERD, Clostridium difficile infection, microbiome, pre-post intervention trials, allergic diseases

We are writing this commentary to discuss Castellani et al's clinical intervention trial of esomeprazole treatment for gastroesophageal reflux disease (GERD) in infants, particularly in view of the recent large-scale study by Miter et al. that reported a ~1.5-fold greater risk of allergic disease among young infants receiving proton pump inhibitors (Castellani et al., 2017; Mitre et al., 2018). Proton pump inhibitors (PPIs) are a group of medications commonly prescribed in adults and increasingly, in pediatric populations. Essentially, they increase the pH of the gastrointestinal tract, preventing undesirable symptoms related to gastric acidity such as vomiting and pain. In addition to allergic outcomes, other adverse side effects have also been reported with PPI treatment, notably increased risk for intestinal *Clostridium difficile* infection (CDI). In a meta-analysis of 23 studies, PPI use was associated with a 65% increase in CDI risk (Janarthanan et al., 2012). Gut microbial dysbiosis with other taxa has also been reported following PPI treatment (Freedberg et al., 2015a; Shin et al., 2016; Naito et al., 2018) and is characterized by the overgrowth of *Streptococcus* spp., *Enterococcus* spp., and *Escherichia* spp. (Imhann et al., 2016; Takagi et al., 2018).

Castellani et al. are the first to report on the gut microbiome in relation to PPI treatment of infants with a confirmed diagnosis of GERD (Castellani et al., 2017), concluding that esomeprazole treatment of 12 study infants was not associated with significant alteration to gut microbial composition. In particular, neither diversity nor mean relative abundances of the bacteria characteristic of PPI-related dysbiosis (i.e., *Streptococcus* spp.) were significantly different between stool samples collected before and during PPI treatment. PPI's are also prescribed to healthy infants without confirmed GERD—almost 5% of infants in the CHILD (Canadian Healthy Infant Longitudinal Development) cohort received a PPI before they reached the age of 3 months. Hence, we wish to further comment on the findings presented by Castellani et al. and identify issues with their interpretation.

Castellani et al. conducted a pre-post intervention design to test the efficacy and adverse effects of an ~18-week treatment course of esomeprazole, a study design that has major limitations for studying the developing infant gut microbiome. This is because microbial diversity of the infant gut naturally increases with advancing age and in response to feeding practices (Bäckhed et al., 2015), as was shown in the Castellani paper following the discontinuation of PPI therapy. While Castellani et al. correctly identified age and feeding status as confounding factors, they failed to comment on the noticeably absent expansion of infant gut microbial diversity during the initial 4 weeks of esomeprazole treatment. We propose that this lack of significant increase to microbial diversity is a consequence of the PPI medication itself (Figure 1). Our hypothesis is supported by many studies in adults that report lower gut microbial richness and diversity with PPI use (Imhann et al., 2016; Jackson et al., 2016). Thus, a requisite addition to the pre-post study design would have been a control group of healthy untreated infants. Once matched on age, birth mode and feeding status, it is unlikely that the microbiome of a control group would be more heterogeneous than that of the infants included in the Castellani study.

**Figure 1:**
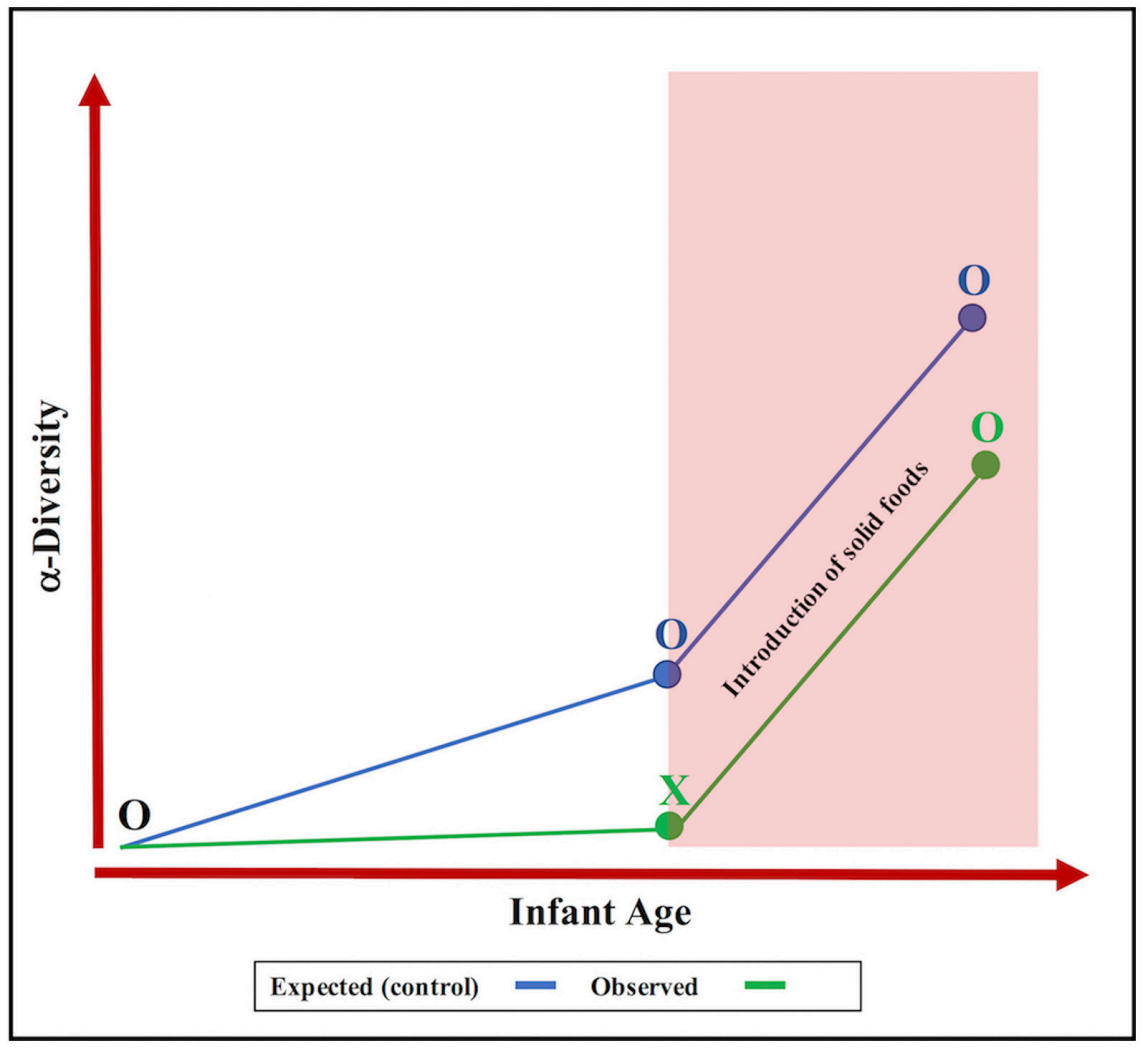
The overlooked effect of proton pump inhibitors on the development of the infant gut microbiota. Expected progression (blue line) vs. observed progression (green line) shows that PPI intervention (X) may inhibit the expansion of α-diversity (O = no treatment).

Furthermore, Castellani and colleagues were hesitant to discuss trends supporting PPI-induced dysbiosis (higher abundance of *Streptococcus* and *Enterococcus* species, reduced *Clostridiaceae*), which may have reached statistical significance with a larger sample size. These compositional changes are evident in several studies of adult PPI use (Freedberg et al., 2015b), including the ones we cited above. Recognizing that PPI-associated changes to the adult fecal microbiome may be more pronounced, consistency between infants and adults in the nature of observed compositional changes to individual microbial species are worth emphasizing. An upward trend in the abundance of *Streptococcus* spp. was noticeable among esomeprazole-treated infants despite a small sample size (Castellani et al., 2017). In fact, streptococci also become more abundant in the oral microbiome following esomeprazole treatment which continues to support the universal and concerning impact of these medications on the microbiota (Mishiro et al., 2018).

Finally, although the authors commented on the influence of age, they did not report the individual ages of the 12 participating infants. It appears that the study population is biased toward an older cohort (>6 months of age) because 11 of 12 infants were fed solid foods by the end of the study period. Additionally, all study participants had a proven diagnosis of GERD. Meanwhile, the peak incidence of “naturally-occurring” gastroesophageal reflux symptoms occurs at ~4 months after birth due to immature esophageal musculature (Martin et al., 2002; Lightdale and Gremse, 2013). As such, we point to the possibility of “indication creep” for the PPI treatment of infant colic and spitting symptoms against a backdrop of insufficient evidence of drug efficacy (Gieruszczak-Białek et al., 2015). Expansion of PPI use may be accompanied by a real danger of disrupted development of the infant gut microbiome and subsequently, future allergic disease. In closing, we applaud Castellani and colleagues for implementing an assessment of the risk of gut microbiome dysbiosis in a trial of PPI treatment in infants. However, such a study requires the inclusion of appropriate control subjects to discern the effect of PPI use on the colonization and early development of the infant gut microbiome.

## Author Contributions

KMD drafted and revised the manuscript. ALK initiated and revised the manuscript. HMT provided feedback and suggestions. All authors approved the final version of the manuscript for submission.

### Conflict of Interest Statement

The authors declare that the research was conducted in the absence of any commercial or financial relationships that could be construed as a potential conflict of interest.
